# In Situ Characterization of the Initial Effect of Water on Molecular Interactions at the Interface of Organic/Inorganic Hybrid Systems

**DOI:** 10.1038/srep45123

**Published:** 2017-03-22

**Authors:** Sven Pletincx, Lena Trotochaud, Laura-Lynn Fockaert, Johannes M. C. Mol, Ashley R. Head, Osman Karslıoğlu, Hendrik Bluhm, Herman Terryn, Tom Hauffman

**Affiliations:** 1 Department of Materials and Chemistry, Research Group Electrochemical and Surface Engineering (SURF), Vrije Universiteit Brussel, Pleinlaan 2, 1050 Brussels, Belgium; 2 Chemical Sciences Division, Lawrence Berkeley National Laboratory, Berkeley, CA 94720, United States of America; 3 Department of Materials Science and Engineering, Delft University of Technology, Mekelweg 2, 2628 CD, Delft, The Netherlands

## Abstract

Probing initial interactions at the interface of hybrid systems under humid conditions has the potential to reveal the local chemical environment at solid/solid interfaces under real-world, technologically relevant conditions. Here, we show that ambient pressure X-ray photoelectron spectroscopy (APXPS) with a conventional X-ray source can be used to study the effects of water exposure on the interaction of a nanometer-thin polyacrylic acid (PAA) layer with a native aluminum oxide surface. The formation of a carboxylate ionic bond at the interface is characterized both with APXPS and *in situ* attenuated total reflectance Fourier transform infrared spectroscopy in the Kretschmann geometry (ATR-FTIR Kretschmann). When water is dosed in the APXPS chamber up to 5 Torr (~28% relative humidity), an increase in the amount of ionic bonds at the interface is observed. To confirm our APXPS interpretation, complementary ATR-FTIR Kretschmann experiments on a similar model system, which is exposed to an aqueous electrolyte, are conducted. These spectra demonstrate that water leads to an increased wet adhesion through increased ionic bond formation.

Stability of bonds between carboxylic acid functional groups of a polymer and a hydroxylated surface of aluminum oxide in aqueous and corrosive environments is of pivotal importance to a broad range of applications^1^,^2^,^3^,^4^,^5^,^6^. However, the inevitable presence of defects in these coatings allows water and ions to penetrate into the coating and, with sufficient time, all the way to the polymer/oxide interface^7^,^8^,^9^,^10^. The presence of water and hydroxyls at the polymer/oxide interface region may result in polymer delamination due to the partial or full displacement of interfacial bonds by a more stable interaction between the metal oxide and water. Currently, a “trial-and-error” approach is carried out to develop new coatings and test them for more desirable properties in humid environments. Common and practical techniques to evaluate the durability of coated oxide samples are typically accelerated industrial tests (e.g. salt spray, exposure to UV light, or mechanical tests)^11^,^12^,^13^,^14^. These tests are relevant for macroscopic failure and overall performance analysis. However, during these industrial tests, multiple processes likely occur simultaneously at the polymer/metal oxide interface, leading to failure of the coating. For example, salt-spray tests not only involve exposure of the coating to changing humidity conditions, but corrosion processes which can involve highly localized potential gradients and multiple chemical pathways may be complicit in coating delamination. Therefore, macroscopic industrial tests lack the ability to provide detailed information on the chemical interfacial bonding (failure) mechanism. Conventional chemical analysis techniques (e.g. X-ray photoelectron spectroscopy, Auger electron spectroscopy) can provide information on the surface chemistry of metal (oxides). However, these techniques typically do not permit analysis under atmospheric conditions and cannot prevent changes of *ex situ* applied surface chemistry modifications upon exposure of the sample to an ultra-high vacuum environment.

Recent developments in the field of ambient-pressure X-ray photoelectron spectroscopy (APXPS) enable a novel approach to probe the chemical state of these interfaces^15^,^16^. A broad range of relative humidity (RH) conditions can be reached in the analysis chamber, making it possible to unravel interfacial chemistry changes of organic/inorganic systems *in situ*. In order to access the buried interface, a thin-film approach is used: this approach was introduced by Watts *et al*.^17^ and was successfully applied to polymethyl methacrylate (PMMA)^18^,^19^ and polyacrylic acid (PAA)^20^ on various metal oxides. Observed changes in the C 1s core level in *ex situ* XPS measurements revealed interfacial acid-base interactions of the functional groups of the polymer with the metal oxide surfaces. Later, this thin-film approach was adopted by Alexander *et al*.^21^ and other groups^22^,^23^,^24^,^25^ to investigate the interaction of carboxylic acid functional groups on metal oxides. Several studies using *ex situ* XPS^18^,^20^,^21^,^26^,^27^,^28^ and conventional ATR-FTIR^1^,^29^,^30^,^31^ have shown the existence of carboxylate anion bonds, as a result of a reaction of the carboxylic acid functional groups with the surface hydroxyl groups of the aluminum oxide surface.

We have implemented the thin-film approach here by applying a sufficiently thin film (~3 nm) of PAA such that photoelectrons from the polymer/oxide interface region are able to escape the interface to be detected by APXPS. The synthesized hybrid system is then exposed to water vapor in the analysis chamber, which makes it possible to follow initial changes at the interface *in situ*. To confirm the trends observed by APXPS, ATR-FTIR in the Kretschmann geometry is utilized^32^,^33^,^34^,^35^. This technique allows an interface-sensitive and *in situ* analysis of a polymer/metal oxide interface. In this study, we first use the ATR-FTIR Kretschmann technique to characterize the formed bonds at the interface *in situ* by reactive adsorption for 24 hours. Then, this technique was used to study the effect of an aqueous electrolyte at the PAA/aluminum oxide interface. In both the APXPS and Kretschmann ATR-FTIR experiments, the choice of an appropriate model sample system is critical for application of the techniques to these polymer/oxide hybrid interfaces in a complementary manner. For the APXPS experiments, a model coating is applied on a technical metal oxide surface, but the humidity exposure is limited by the technique. For the ATR-FTIR Kretschmann experiments, the coating is applied on a model metal oxide, which is then exposed to an excess of water. The use of these dedicated model systems allows us to focus only on humidity-induced changes to interfacial bonding, offering a unique advantage by eliminating other complex processes that might occur during macroscopic failure tests.

## Experimental

### Materials and sample preparation

The metal substrates used for the APXPS study were cut from a 0.3 mm ultrapure aluminum sheet (99.99% metals basis, rolled sheet, Norsk Hydro Aluminium). Samples were rinsed ultrasonically in acetone for 5 min. After cleaning, the samples were chemically etched in a 25 g L^−1^ NaOH solution. Then the samples were blown dry with nitrogen and exposed to atmosphere to let natural oxide growth occur. Thin polymer films were deposited by reactive adsorption on the substrate from a 0.01% w/w PAA (Mw = 450.000 g mol^−1^) solution in methanol. After 24 h, the samples were removed from the solution and placed in pure solvent to remove physisorbed polymer chains so that all remaining chains are chemisorbed to the oxide surface.

For the ATR-FTIR in Kretschmann geometry measurements, a germanium internal reflection element (IRE) hemisphere was coated with pure aluminum (99.99% metals basis, pellets, Johnson Matthey) by means of a high-vacuum evaporation coating system (Balzers BAE 250). The film thickness was around 50 nm, as determined with a quartz crystal microbalance. The metal film was exposed to ambient conditions to form a native oxide layer. On top of this thin metal oxide film, the polymer coating was deposited by placing the polymer solution in the Seagull cell and measuring *in situ* the adsorption at the interface. A spectrum of the IRE crystal coated with aluminum oxide against pure methanol was taken as a background prior to the polymer deposition and polymer solution exposure.

For the water ingress measurements, the polymer was deposited by drop casting of the 0.01% w/w PAA/methanol solution. The electrolyte used in the Seagull cell, was a 0.1 M borate buffer to investigate the effect of water at the interface. A spectrum of the IRE crystal coated with aluminum oxide against ambient conditions was taken as a background prior to the polymer deposition and prior to electrolyte exposure.

### Interface Characterization

#### Ambient pressure X-ray photoelectron spectroscopy (APXPS)

Samples were introduced into the analysis chamber of the APXPS (SPECS Phoibos 150 NAP) immediately after casting the thin film. A glass vessel containing water (18.2 MΩ cm) was mounted onto the APXPS analysis chamber, degassed by three cycles of liquid nitrogen freeze-pump-thaw, and water vapour was introduced into the XPS analysis chamber via a precision leak valve. All core levels were acquired using a pass energy of 20 eV and an energy step of 0.05 eV. The X-ray source (Al K-alpha, monochromatized, SPECS MF 60) was set at an acceleration voltage of 14 kV and an irradiation power of 100 W was used. For the fitting procedure the software KolXPD was used. The peak shape is a mixed Gaussian-Lorentzian, with a Shirley type background. After curve fitting, the binding energy scale was calibrated by setting the lowest binding energy component of the C 1 s peak to 284.8 eV.

#### Fourier transform infrared spectroscopy in the Kretschmann geometry (ATR-FTIR Kretschmann)

A Thermo-Nicolet Nexus Fourier transform infrared spectroscopy (FTIR) apparatus equipped with a mercury−cadmium−telluride (MCT) liquid-nitrogen-cooled detector, and a nitrogen-purged measurement chamber with a Harrick Seagull multipurpose reflection accessory was used. The resolution of the acquired spectra is 8 cm^−1^. Spectra acquisition was controlled by the OMNIC 8.1 software package (ThermoElectron Corporation, Madison, WI).

## Results and Discussion

### APXPS measurements of an ultrathin PAA film on aluminum oxide, exposed to different water vapour pressures

**Figure 1:**
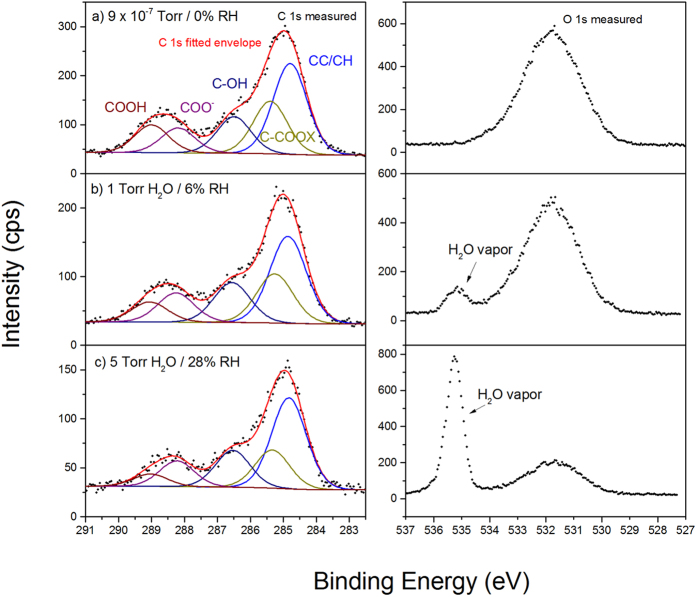
C 1s (left) and O 1s (right) APXPS spectra of an ultrathin PAA film on native aluminum oxide at varying water vapor pressures. 9 × 10^−7^ Torr (**a**), 1 Torr H_2_O (**b**), 5 Torr H_2_O (**c**).

The coated samples were investigated under three conditions: as-prepared and under UHV, at low relative humidity (6% RH), and at a higher relative humidity (28% RH). The higher relative humidity condition corresponds to the highest pressure (5 Torr) that could be reached in the analysis chamber before strong attenuation of the signal by the vapour phase occurs. In Fig. 1, the C 1s (left) and O 1s (right) spectra at different water vapour pressures are shown together with the peak fits of the corresponding C 1s spectra. In both the C 1s and O 1s spectra, it can be seen that the peak intensities (plotted here in cps after Shirley-background subtraction) for surface species are attenuated as the water vapour pressure is increased. The C 1s spectra is fit with components assigned to C-C/C-H, **C**-COOX, C-OH, COO^−^, and COOH species. In the O 1s spectra, the water vapour phase peak is observed at higher BE than the contributions from the surface species, which appear together as a broad feature containing contributions from aluminum oxide, PAA, and (after exposure to water vapour) surface hydroxyls and/or adsorbed water. The individual components are not well resolved in these O 1s spectra, making fitting these spectra quite complex. Thus, we focus here on interpretation of the C 1s spectra.

Following the previously suggested peak fitting strategy for thin PAA films on metal oxides by Leadley *et al*.^20^ and Alexander *et al*.^26^, two additional C 1s peaks are required during fitting in comparison with the spectrum of the bulk polymer^36^. Bulk PAA has three main peaks in the C 1s peak: C-C/C-H (284.8 eV), **C**-COOX (+0.4 eV BE shift with respect to C-C/C-H), COOH (+4.2 eV BE shift with respect to C-C/C-H). The first additional peak is fitted at +1.7 eV with respect to the C-C/C-H peak and corresponds to C-OH species. This peak was previously assigned to ether functionalities created by X-ray damage^20^. In this work under the X-ray flux used, we did not observe any significant changes in XPS spectra due to beam exposure (shown in SI). Another suggestion was that end-groups of the polymer are more prominent when an ultrathin film is applied^26^. FTIR measurements before water exposure (as shown later), show that OH groups are already present at the interface, since a peak at 3426 cm^−1^ is observed. We assign part of this FTIR peak at 3426 cm^−1^, together with the observed C-OH peak in the APXPS data, to the presence of residual solvent (methanol) in the polymer. We propose that some methanol interacts strongly with the aluminum oxide surface (i.e. it is not simply physisorbed). Chemisorption of methanol molecules with the aluminum oxide surface were observed in other investigations, which show oxygen lone pair interactions^37^,^38^. This explains why this peak remains visible in APXPS spectra even at UHV conditions, since any free solvent molecules would otherwise be expected to evaporate due to the low pressure in the analysis chamber.

The second additional peak in the C 1s fit is the carboxylate peak (COO^−^), which is the ionic bond formed at the hybrid interface and has a BE shift of +3.4 eV relative to the aliphatic carbon peak. This BE shift is in accordance with previous measurements that give values between +3.2 and +3.5 eV^18^,^19^,^20^,^21^,^26^. In order to fit the intensity of the higher BE component, the intensity of the beta-shifted carbon peak (**C**-COOX) was constrained to equal the sum of the carboxylate (COO^−^) and carboxylic acid (COOH) components, as each COOH and COO^−^ should have one adjacent beta-carbon based on the structure of the polymer. We note that the intensity of the C-C/C-H peak is somewhat larger than would be expected for PAA based on the intensity of the **C**-COOX peak (ratio expected from PAA structure is 1:1). In previous investigations, this increased C-C/C-H intensity was attributed to an excess of hydrocarbons from the polymer itself^21^,^26^,^36^. However, in these cited works ambient-borne contamination was not taken into account. The manufacturer of the polymer used in this work states that only benzene could occur as an impurity with a concentration smaller than 0.1%. Due to this knowledge about the polymer impurity and since it is known that surface carbon contamination is typically removed during the PAA deposition process^29^,^31^,^39^,^40^, we attribute this extra intensity to aliphatic adventitious carbon *on top* of the polymer film. It was estimated by Koo *et al*. that the coverage of ambient-borne contaminants ranged between 5–10% of the uncoated substrate and it was observed that this contamination was rapidly displaced by polymer molecules at the aluminum oxide surface by immersion in the PAA solution^40^. Since this is a small contribution to the surface species and is not primarily located at the polymer/oxide interface, we assume that the effects of adventitious carbon on the interface chemistry are negligible.

**Figure 2:**
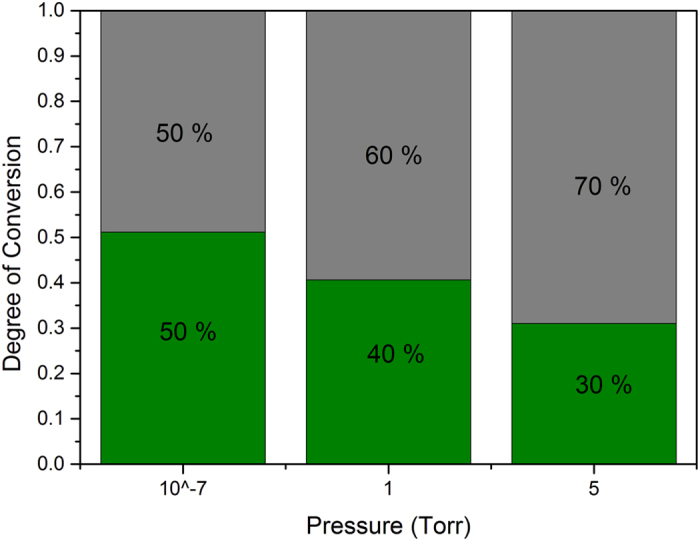
Degree of conversion of the carboxylic acid group at different H_2_O pressures in the APXPS chamber. Grey: [COO^−^]/([COO^−^] + [COOH]); Green: [COOH]/([COO^−^] + [COOH]).

When the pressure in the analysis chamber is increased by dosing water, the relative intensity of the carboxylate peak increases whereas that of the carboxylic acid peak decreases. Relative intensities of the other C 1s peaks show little changes and no BE shifts occur during water exposure. It is proposed that this indicates a deprotonation of the carboxylic acid groups by water forming the carboxylate groups, followed by ionic bond formation with the surface hydroxyl groups of the aluminum oxide. This was also suggested by Tannenbaum *et al*.^30^ when they were studying the adsorption of acrylic polymers on alumina surfaces by infrared spectroscopy. Tannenbaum *et al*. propose that the bonding with the aluminum oxide sites is mediated by the presence of water. The observations made in our work show that the presence of water indeed leads to more adsorption of the functional groups to the surface of the oxide. The degree of conversion of carboxylic acid to carboxylate is calculated as follows from the XPS peak areas: [COO^−^]/([COO^−^] + [COOH]). A value of 0.5 is obtained at 9 × 10^–7^ Torr. This value is in accordance with other work where values of 0.4 were observed^26^. When water is introduced and the pressure increases to 1 Torr the degree of conversion is 0.6. At a pressure of 5 Torr H_2_O the degree of conversion reaches a value of 0.7 as shown in Fig. 2. The fact that there is not full conversion in the thin polymer layer has multiple reasons, including that due to the high molecular weight, the functional groups are sterically hindered.

### *In situ* ATR-FTIR of the interface between native aluminum oxide and a 0.01 wt% PAA/methanol solution

**Figure 3:**
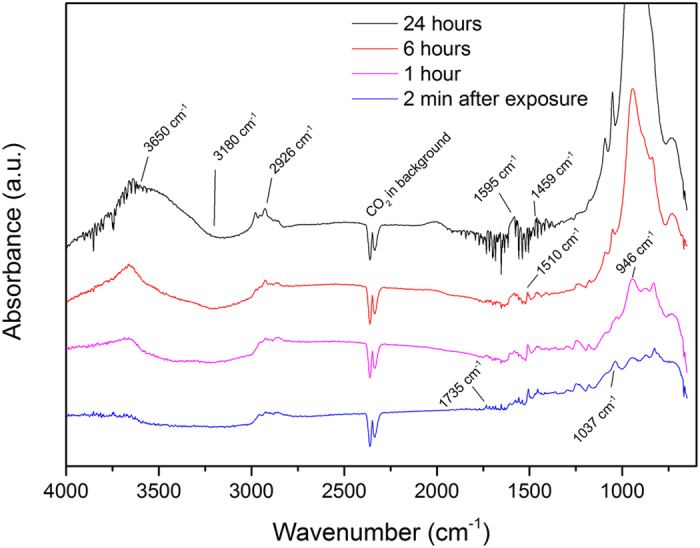
ATR-FTIR Kretschmann spectra of a PAA/methanol solution on native aluminum oxide. Spectra were taken at different times to follow the adsorption of the polymer on the aluminum oxide surface *in situ*.

*In situ* ATR-FTIR measurements of the oxide/polymer interface were performed on the aluminum oxide coated IRE crystal during exposure to a PAA polymer solution in methanol. In Fig. 3, the interface spectra are shown at different adsorption (i.e. solution-exposure) times. The oxide-coated IRE crystal in contact with pure methanol (no PAA in solution) was used as the background measurement and is subtracted from the spectra shown in Fig. 3. Although the cell was purged with N_2_ gas, the effect of a variable background atmosphere can be seen in the spectrum around the regions 4000–3500 cm^−1^ and 2000–1250 cm^−1^ taken after 24 hours; this wave pattern is typical for a change in the concentration of water vapour in the cell. The spectra show a downward peak at 3180 cm^−1^ that increases in intensity with increasing exposure time. This peak, which is observed at a lower wavenumber than is typical for free OH-groups, corresponds to the disappearance of hydrogen-bonded methanol at the interface. Peaks around 2926 cm^−1^ are also observed, which correspond to CH_2_ stretching vibrations, and display increasing intensities over time. This indicates that the solvent methanol is being replaced by the polymer at the oxide/solution interface. The carbonyl vibration band (C=O) is seen at 1735 cm^−1^. The much lower intensity of this peak with respect to other peaks shows the power of the ATR-FTIR technique in Kretschmann configuration, since it is known that the bulk spectra of PAA contains a large carbonyl band (see SI). This implies that the spectra are interface specific and that some carbonyl groups of the COOH functional groups are present at the interface but their density is much less at the interface than in the bulk of the polymer. The presence of bands around 1450–1600 cm^−1^ indicate that carboxylate species are coordinatively bonded to the aluminum oxide. The peak at 1595 cm^−1^ is assigned to the ν_as_(COO^−^) carboxylate stretch^24^. Features for the symmetric carboxylate stretch ν_s_(COO^−^) can be found at 1459 cm^−1^ and 1510 cm^−1^. Additionally to the observation of the ionic bond, it is seen that the carbonyl stretch at the interface (C=O) is observed at lower wavenumber (1735 cm^−1^) than for free bulk carbonyl stretches that can typically be found around 1750 cm^−1^ 41. This downward shift indicates hydrogen bonding where the carbonyl oxygen acts as the Lewis base and the proton of the hydroxyl group is the Lewis acid^42^.

The peaks observed at 946 cm^−1^ and 1037 cm^−1^ are from the Al-O and Al-OH stretching and bending vibrations. It has been shown previously by van den Brand *et al*.^43^ that water adsorbs on the aluminum oxide surface (which cannot be removed by solvents) and causes growth and hydroxylation of the oxide layer. This explains the increase over time of these Al-O and Al-OH peaks. Also over time, an increasing OH peak at 3650 cm^−1^ is observed, which is consistent with the increase of the Al-O and Al-OH peak.

These *in situ* adsorption spectra show that ionic bonds are formed at the PAA/aluminum oxide interface over the course of 24 hours and that the reactive adsorption deposition method used here is successful in creating a chemically-bound PAA layer.

### The effect of water at the PAA/aluminum oxide interface followed by ATR-FTIR Kretschmann

**Figure 4:**
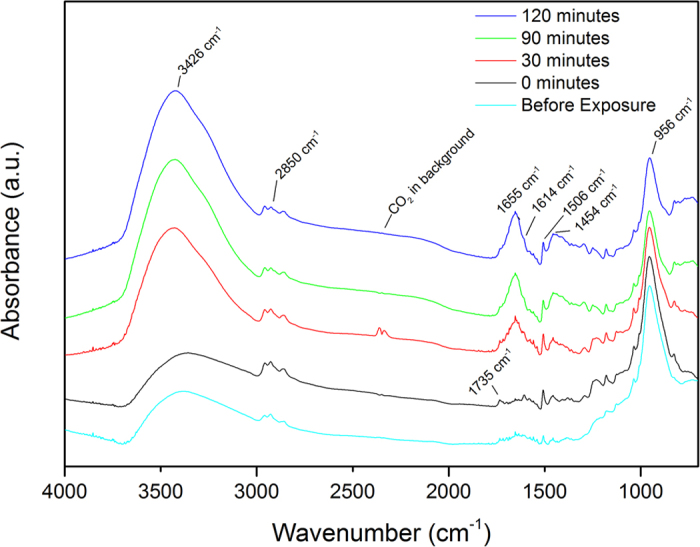
ATR-FTIR Kretschmann spectra of PAA on native aluminum oxide. Spectra were taken at different times before and after exposure to a 0.1 M borate buffer solution.

**Figure 5:**
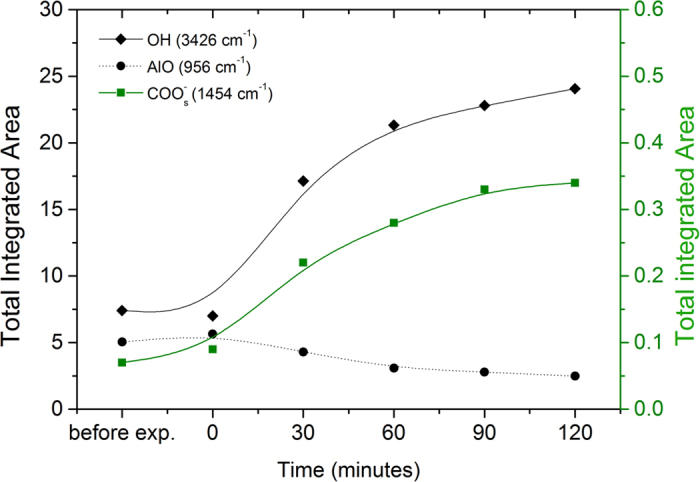
Infrared peak areas of 0.01 wt% PAA on native aluminum oxide before and after exposure of water on the polymer coating as a function of time after the start of exposure. ν(OH): 3426 cm^−1^; ν(Al-O): 956 cm^−1^ and ν_s_(COO^−^): 1454 cm^−1^.

As a complementary technique to APXPS, ATR-FTIR in Kretschmann geometry was also used to study the effect of water on the hidden interface between the polymer/metal oxide system. This vibrational spectroscopic technique is interface-sensitive and allows for *in situ* monitoring of the uptake of an electrolyte through the polymer coating and the resulting chemical changes at the buried interface. However, it must be mentioned that the deposited metal oxide by evaporation (as used in this experiment) has different metallurgical properties than a rolled sheet of the metal with a native oxide surface (as used in our XPS experiments). Therefore, this technique can be considered to provide information on the chemical changes at a model polymer/metal oxide interface mimicking a real system of interest. The results obtained with this technique verify the existence of the species which correspond to the peaks used to fit the APXPS data. In Fig. 4, the IR spectra are shown of a 0.01 wt% PAA adsorbed on an aluminum oxide coated germanium crystal when a 0.1 M borate buffer was used as an electrolyte. The IR background was that of an aluminum oxide coated germanium crystal, prior to polymer deposition in contact with ambient air. When considering the spectrum before any electrolyte has been introduced to the system, the carbonyl vibration band (C=O) is seen at 1736 cm^−1^. The presence of bands around 1450–1600 cm^−1^ indicate that carboxylate species are formed at the interface, providing justification for fitting a carboxylate peak in the C 1s XPS spectra. The spectra taken before exposure to the electrolyte shows a peak at 3426 cm^−1^, which is assigned partly to the OH group of interacting methanol molecules and partly to the hydroxyl groups of the aluminum oxide surface due to ageing of the substrate surface in contact with the ambient atmosphere. The peak at 1614 cm^−1^ is assigned to the ν_as_(COO^−^) carboxylate stretch^24^. Features for the symmetric carboxylate stretch ν_s_(COO^−^) can be found at 1454 cm^−1^ and 1506 cm^−1^. The peak at 956 cm^−1^ is assigned to the ν(Al-O) of free surface hydroxyl groups on aluminum oxide^41^,^43^,^44^. The bands between 3000–2800 cm^−1^ are assigned to CH_2_ stretching, indicating the presence of CH_2_ of the polymer chain at the interface. In Fig. 5, the integrated absorbance of several peaks are plotted before and at every 30 minutes after exposure to the electrolyte, up to a total exposure time of 120 minutes.

When the electrolyte is added to the cell, the OH peak increases at 3426 cm^−1^ and the symmetric carboxylate peak increases in a similar trend. This is consistent with the observations from APXPS measurements as discussed previously. At the same time, the Al-O peak decreases at the interface, which is consistent with the hypothesis that hydroxyls engage with the newly formed carboxylate anion groups in order to form ionic bonds. Another decrease is observed in the CH_2_ peak, which can be explained by H_2_O and ionic bonds replacing this group at the interface.

### Proposed adsorption mechanism of PAA on aluminum oxide

**Figure 6:**
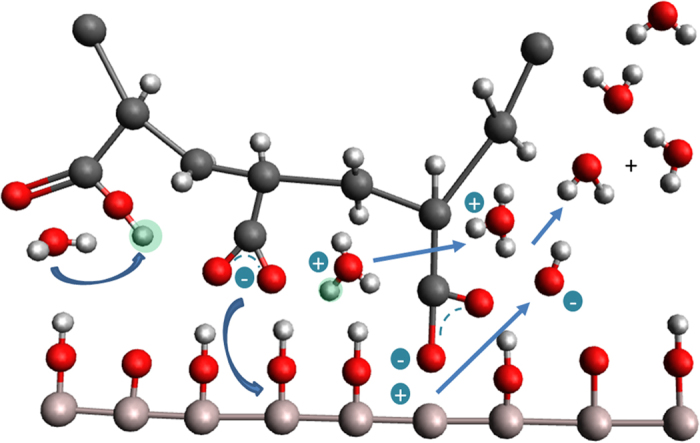
Schematic representation of proposed adsorption mechanism. Polyacrylic Acid on aluminum with an oxide/hydroxide surface in the presence of water at the hybrid interface. Water has a mediating role in the formation of ionic bonds at the interface.

We propose an adsorption mechanism, shown in Fig. 6, of several equilibrium reactions that take place at the interface. Water has a mediating role at the interface, where it deprotonates the carboxylic acid functional groups creating hydroxonium ions. The formed carboxylate species react with the surface hydroxyl creating an hydroxide ion, which together with the previously formed hydroxonium ion sustains the self-ionization water equilibrium. More water at the interface leads to more carboxylate species and thus more interfacial bonds up till the point where all the available functional groups are bonded. At this point, water will replace the formed ionic bond, which will eventually lead to macroscopic delamination. No evidence of delamination was observed in XPS experiments, suggesting that the delamination point is at a relative humidity greater than 28% RH (this corresponds to water vapour pressure of 5 Torr, which is the highest pressure accessible in the APXPS device).

## Conclusion

In summary, our investigations reported here probe the effect of water on the interfacial interactions of a PAA/aluminum oxide interface *in situ.* It was shown that APXPS can be used to identify an ionic bond at this interface, and that the amount of these bonds increases when the water pressure in the analysis chamber is increased up to a RH of 28%. Complementary to this technique, *in situ* ATR-FTIR in the Kretschmann geometry was utilized to characterize the formed bonds during reactive adsorption and to describe the effect of water on the interface. This technique also shows that the amount of carboxylate ionic bonds increases when water was introduced. The observations made with both techniques allow us to propose a bonding mechanism of carboxylic acid functional groups when water is present at the interface. Water plays a mediating role at the interface, where the carboxylic acid functional groups are deprotonated to form carboxylate ions and hydronium ions. The carboxylate ions then react with the surface hydroxyl groups of the aluminum surface to form ionic bonds and hydroxide ions. The formed hydronium and hydroxide ions sustain the self-ionization equilibrium of water.

## Additional Information

**How to cite this article**: Pletincx, S. *et al*. In Situ Characterization of the Initial Effect of Water on Molecular Interactions at the Interface of Organic/Inorganic Hybrid Systems. *Sci. Rep.* **7**, 45123; doi: 10.1038/srep45123 (2017).

**Publisher's note:** Springer Nature remains neutral with regard to jurisdictional claims in published maps and institutional affiliations.

## Supplementary Material

Supplementary Information

## References

[b1] van den Brand J., Blajiev O., Beentjes P. C. J., Terryn H. & de Wit J. H. W. Interaction of anhydride and carboxylic acid compounds with aluminum oxide surfaces studied using infrared reflection absorption spectroscopy. Langmuir 20, 6308–6317 (2004). 15248717 10.1021/la0496845

[b2] Treverton J. A., Ball J. & Fairlie M. The reactions of oxidised aluminum surfaces with lubricant additives and related compounds. Appl. Surf. Sci. 52, 107–124 (1991).

[b3] Laibinis P. E., Hickman J. J., Wrighton M. S. & Whitesides G. M. Orthogonal Monolayers: Carboxylic Alumina. Science (80-.). 245, 4–6 (1989). 10.1126/science.245.4920.845 17773361

[b4] Deck P., Moon M. & Sujdak R. Investigation of fluoroacid based pretreatments on aluminium. Surf. Coatings Int. 81, 478–484 (1998).

[b5] Alexander M. R. . Functionalized plasma polymer coatings for improved durability of aluminium – epoxy adhesive joints: fractography. Surf. Interface Anal. 20, 16–20 (2000).

[b6] Taheri P. . Molecular Interactions of Electroadsorbed Carboxylic Acid and Succinic Anhydride Monomers on Zinc Surfaces. J. Phys. Chem. C 17054–17067 (2011).

[b7] Posner R., Wapner K., Stratmann M., Grundmeier G. & Titz T. Transport processes of hydrated ions at polymer/oxide/metal interfaces. Electrochim. Acta 54, 891–899 (2009).

[b8] Jr H. L., Wang W. & Igetoft L. The mechanism for the cathodic delamination of organic coatings from a metal surface. Prog. Org. Coatings 11, 19–40 (1983).

[b9] Nguyen T., Byrd E., Bentz D. & Lint C. *In situ* measurement of water at the organic coating/substrate interface. Prog. Org. Coatings 27, 181–193 (1996).

[b10] Posner R., Ozcan O. & Grundmeier G. In Design of Adhesive Joints Under Humid Conditions (eds da Silva L. F. M. & Sato C.) 25, (Springer Berlin Heidelberg, 2013).

[b11] Van Den Brand J., Van Gils S., Terryn H., Sivel V. G. M. & De Wit J. H. W. Changes in epoxy-coated aluminium due to exposure to water. Prog. Org. Coatings 51, 351–364 (2004).

[b12] Lane M. W., Snodgrass J. M. & Dauskardt R. H. Environmental Effects on Interfacial Adhesion. Microelectronics Reliability 41, 1615–1624 (2001).

[b13] Rattana A., Abel M.-L. & Watts J. F. Degradation of Interfacial Chemistry of Epoxy/Silane/Aluminium Interfaces as a Result of Aqueous Attack. J. Adhes. 81, 963–988 (2005).

[b14] Özkanat Ö., de Wit F. M., de Wit J. H. W., Terryn H. & Mol J. M. C. Influence of pretreatments and aging on the adhesion performance of epoxy-coated aluminum. Surf. Coatings Technol. 215, 260–265 (2013).

[b15] Ogletree D. F., Bluhm H., Hebenstreit E. D. & Salmeron M. Photoelectron spectroscopy under ambient pressure and temperature conditions. Nucl. Instruments Methods Phys. Res. Sect. A Accel. Spectrometers, Detect. Assoc. Equip. 601, 151–160 (2009).

[b16] Starr D. E., Liu Z., Hävecker M., Knop-Gericke A. & Bluhm H. Investigation of solid/vapor interfaces using ambient pressure X-ray photoelectron spectroscopy. Chem. Soc. Rev. 42, 5833 (2013). 23598709 10.1039/c3cs60057b

[b17] Watts J. F., Chehimi M. M. & Gibson E. M. Acid-Base Interactions in Adhesion: The Characterization of Surfaces & Interfaces by XPS. J. Adhes. 39, 145–156 (1992).

[b18] Watts J. F., Leadley S. R., Castle J. E. & Blomfield C. J. Adsorption of PMMA on oxidized Al and Si substrates: An investigation by high-resolution X-ray photoelectron spectroscopy. Langmuir 16, 2292–2300 (2000).

[b19] Leadley S. R. & Watts J. F. The use of XPS to examine the interaction of PMMA with oxidised metal substrates. J. Electron Spectros. Relat. Phenomena 85, 107–121 (1997).

[b20] Leadley S. R. & Watts J. F. The use of XPS to examine the interaction of poly(acrylic acid) with oxidised metal substrates. J. Electron Spectros. Relat. Phenomena 85, 107–121 (1997).

[b21] Alexander M. R., Payan S. & Duc T. M. Interfacial interactions of plasma-polymerized acrylic acid and an oxidized aluminium surface investigated using XPS, FTIR and poly(acrylic acid) as a model compound. Surf. Interface Anal. 26, 961–973 (1998).

[b22] Marsh J., Minel L., Barthés-Labrousse M. G. & Gorse D. The nature of the surface acidity of anodised titanium: An XPS study using 1,2-diaminoethane. Appl. Surf. Sci. 99, 335–343 (1996).

[b23] Marsh J., Minel L., Barthes-Labrousse M. G. & Gorse D. Interaction of epoxy model molecules with aluminium, anodised titanium and copper surfaces: an XPS study. Appl. Surf. Sci. 133, 270–286 (1998).

[b24] Taheri P. . Bonding mechanisms at buried interfaces between carboxylic polymers and treated zinc surfaces. J. Phys. Chem. C 117, 2780–2792 (2013).

[b25] Taheri P. . A comparison of the interfacial bonding properties of carboxylic acid functional groups on zinc and iron substrates. Electrochim. Acta 56, 1904–1911 (2011).

[b26] Alexander M. R., Beamson G., Blomfield C. J., Leggett G. & Duc T. M. Interaction of carboxylic acids with the oxyhydroxide surface of aluminium: Poly(acrylic acid), acetic acid and propionic acid on pseudoboehmite. J. Electron Spectros. Relat. Phenomena 121, 19–32 (2001).

[b27] Yamabe H. Stabilization of the polymer-metal interface. Prog. Org. Coatings 28, 9–15 (1996).

[b28] DeKoven B. & Hagans P. XPS studies of metal/polymer interfaces—thin films of Al on polyacrylic acid and polyethylene. Appl. Surf. Sci. 27, 199–213 (1986).

[b29] Konstadinidis K. . Segment level chemistry and chain conformation in the reactive adsorption of poly(methyl methacrylate) on aluminum oxide surfaces. Langmuir 8, 1307–1317 (1992).

[b30] Tannenbaum R., King S., Lecy J., Tirrell M. & Potts L. Infrared study of the kinetics and mechanism of adsorption of acrylic polymers on alumina surfaces. Langmuir 20, 4507–4514 (2004). 15969159 10.1021/la036137v

[b31] Allara D. L. & Nuzzo R. G. Spontaneously organized molecular assemblies. 2. Quantitative infrared spectroscopic determination of equilibrium structures of solution-adsorbed n-alkanoic acids on an oxidized aluminum surface. Langmuir 1, 52–66 (1985).

[b32] Öhman M. & Persson D. An integrated *in situ* ATR-FTIR and EIS set-up to study buried metal-polymer interfaces exposed to an electrolyte solution. Electrochim. Acta 52, 5159–5171 (2007).

[b33] Öhman M. & Persson D. ATR-FTIR Kretschmann spectroscopy for interfacial studies of a hidden aluminum surface coated with a silane film and epoxy I. Characterization by IRRAS and ATR-FTIR. Surf. Interface Anal. 44, 133–143 (2012).

[b34] Taheri P., De Wit J. H. W., Terryn H. & Mol J. M. C. *In situ* study of buried metal-polymer interfaces exposed to an aqueous solution by an integrated ATR-FTIR and electrochemical impedance spectroscopy system. J. Phys. Chem. C 117, 20826–20832 (2013).

[b35] Taheri P. . *In Situ* Study of Buried Interfacial Bonding Mechanisms of Carboxylic Polymers on Zn Surfaces. J. Phys. Chem. C 117, 3374–3382 (2013).

[b36] Beamson G. & Briggs D. High Resolution XPS of Organic Polymers: The Scienta ESCA300 Database (1992).

[b37] Nishimura S. Y., Gibbons R. F. & Tro N. J. Desorption Kinetics of Methanol from Al2O3 (0001) Studied Using Temperature-Programmed Desorption and Isothermal Desorption. J. Phys. Chem. B 102, 6831–6834 (1998).

[b38] Frederick B. G., Aoai G. & Rhodin T. N. Defect structure of clean and chlorinated probed by methanol chemisorption aluminum oxide films. Surf. Sci. 277, 337–350 (2001).

[b39] Allara D. L. & Nuzzo R. G. Spontaneously organized molecular assemblies. 1. Formation, dynamics, and physical properties of n-alkanoic acids adsorbed from solution on an oxidized aluminum surface. Langmuir 1, 45–52 (1985).

[b40] Koo E., Yoon S., Atre S. V. & Allara D. L. Robust, functionalizable, nanometer-thick poly(acrylic acid) films spontaneously assembled on oxidized aluminum substrates: Structures and chemical properties. Langmuir, doi: 10.1021/la104840c (2011). 21381773

[b41] van den Brand J., Blajiev O., Beentjes P. C. J., Terryn H. & de Wit J. H. W. Interaction of Ester Functional Groups with Aluminum Oxide Surfaces Studied Using Infrared Reflection Absorption Spectroscopy. Langmuir 20, 6318–6326 (2004). 15248718 10.1021/la049456a

[b42] Brogly M., Nardin M. & Schultz J. Evidence of Acid-Base Interfacial Adducts in Various Polymer/Metal Systems by IRAS: Improvement of Adhesion. J. Adhes. 58, 263–279 (1996).

[b43] Van Den Brand J., Van Gils S., Beentjes P. C. J., Terryn H. & De Wit J. H. W. Ageing of aluminium oxide surfaces and their subsequent reactivity towards bonding with organic functional groups. Appl. Surf. Sci. 235, 465–474 (2004).

[b44] Van Gils S., Melendres C. A. & Terryn H. Quantitative chemical composition of thin films with infrared spectroscopic ellipsometry: Application to hydrated oxide films on aluminium. Surf. Interface Anal. 35, 387–394 (2003).

