# Supplementary material for: In Situ Characterization of the Initial Effect of Water on Molecular Interactions at the Interface of Organic/Inorganic Hybrid Systems
Source: Sci Rep. 2017 Mar 22;7:45123. doi: 10.1038/srep45123 (PMC5361173; doi:10.1038/srep45123)
Supplement: Supplementary Information [file srep45123-s1.pdf]

In Situ Characterization of the Initial Effect of Water on Molecular Interactions at the Interface of Organic/Inorganic Hybrid Systems

Sven Pletincx [1,2], Lena Trotochaud [2], Laura-Lynn Fockaert [3], Johannes M.C. Mol [3], Ashley R. Head [2], Osman Karslıoğlu [2], Hendrik Bluhm [2], Herman Terryn [1], Tom Hauffman[1,]*

1. Department of Materials and Chemistry, Research Group Electrochemical and Surface Engineering (SURF), Vrije Universiteit Brussel, Pleinlaan 2, 1050 Brussels, Belgium.

2. Chemical Sciences Division, Lawrence Berkeley National Laboratory, Berkeley, CA 94720, United States of America.

3. Department of Materials Science and Engineering, Delft University of Technology, Mekelweg 2, 2628 CD, Delft, The Netherlands.

AUTHOR INFORMATION

Corresponding Author

* Tom Hauffman. Email: thauffma@vub.ac.be

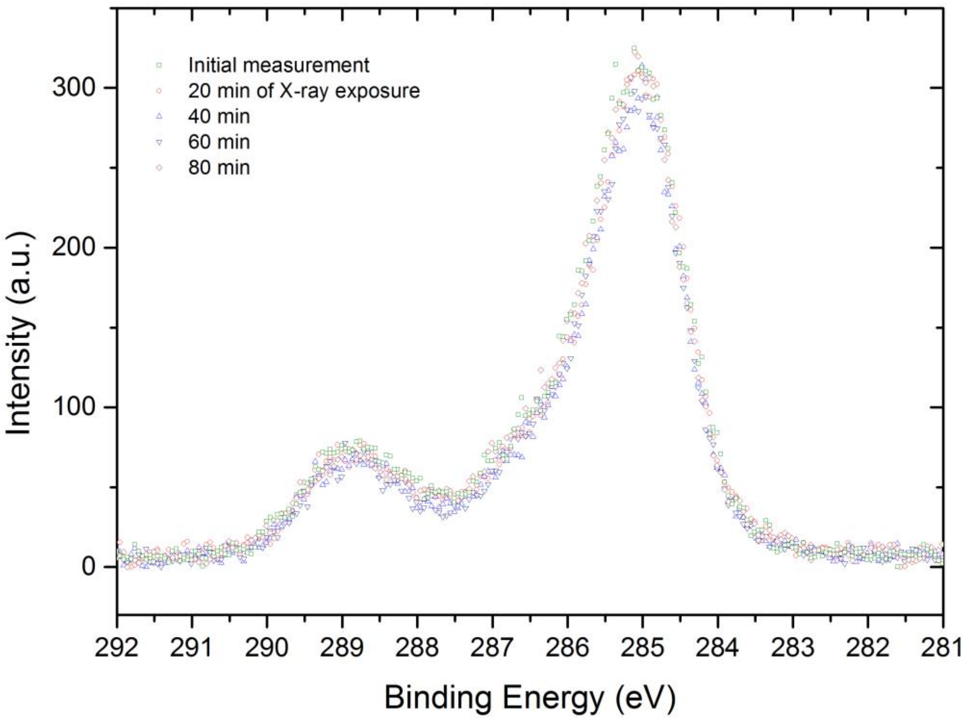

Figure 1. Overlay of APXPS spectra of C 1s peaks of an ultrathin PAA deposition after varying X-ray exposure time. The used source settings were an acceleration voltage of 14 kV and an irradiation power of 100 W. It can be concluded that the used source settings lead to a photon flux at which the polymer does not show x-ray induced damage within 80 minutes of exposure.

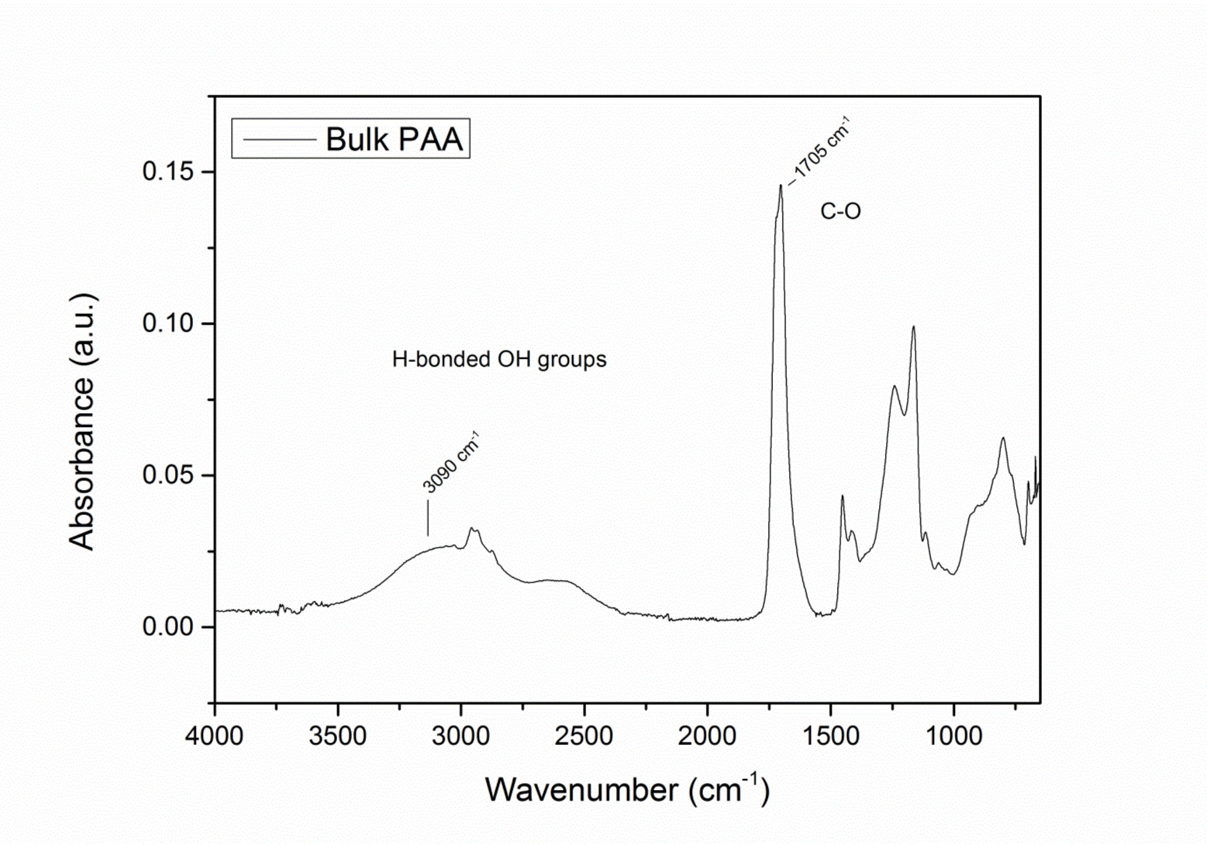

Figure 2. ATR-FTIR spectrum of bulk polyacrylic acid. This spectra was taken with a conventional ATR-FTIR setup on a diamond crystal. Hydroxyl peak at 3090 cm^{-1} corresponds to a hydrogen bonded OH group. Carbonyl group at 1705 cm^{-1}, also found at lower wavenumber indicating the group is hydrogen-bonded. This indicates to interchain hydrogen-bonding.

Peak normalization due to gas phase attenuation

It is important to note that when comparing peaks at different water vapour pressures, the attenuation of the peaks by the gas phase has to be accounted for. This is why a decrease in the raw intensity C-C/C-H (and all the other surface peaks) is observed. Ideally, one would normalize for the signal attenuation from a peak that is known to not be changing upon water vapour exposure. In this case, we are somewhat limited based on the identity of the peaks available in the C 1s spectra. Since the C-C/C-H peak likely contains surface carbon contamination, it is not feasible to normalize the COO^- and COOH areas to this peak. We therefore calculate the ratio by comparing the relative intensity between the COO^- and COOH.

The beta C (**C**-COOX) intensity is set to be the sum of the areas of the two peaks COO^- and COOH, and thus is also not an option for normalization. Furthermore, the ratio of the **C**-COOX and C-C/C-H peaks is always about 0.5 (barring some small changes which could be accounted for by changes in carbon contamination at different spots on the sample) and does not decrease systematically with increasing water vapour pressure. Thus, because of the constraint imposed on the **C**-COOX intensity, the COO^- and COOH peaks are also not systematically decreasing with increasing water vapour pressure.
